# Supplementary material for: Variability of autonomic nerve activity in dry eye with decreased tear stability
Source: PLoS One. 2022 Nov 16;17(11):e0276945. doi: 10.1371/journal.pone.0276945 (PMC9668180; doi:10.1371/journal.pone.0276945)
Supplement: S1 Table — (PDF) [file pone.0276945.s001.pdf]

ID	age	M1F1	systemic disease		OSDI	DEdx	Stress	BUT	F	S	LF0	LF0	LF1	LF2	LF3	LF4
1	39	2	0		18.8	1	10	3.3	1	3	33.46	95.56	181.02	78.78	24.74	28.12
2	63	1	0		8.3	0	13	2.7	0	5	50.27	9.11	10.25	15.5	8.33	69.96
3	54	2	1	HT	16.7	0	21	5.3	0	10	21.9	20.48	32.22	19.75	33.42	13.2
4	42	2	0		25	1	21	3.3	0	4	45.46	24.19	42.09	71.77	37.23	27.79
5	71	2	1	HT	18.8	1	21	4	0	11	29.55	8.36	13.76	63.7	39.84	34.67
6	20	2	0		45	1	22	1	2	8	42.51	65.77	76.74	103.87	116.24	78.47
7	21	2	0		9.1	0	21	4	0	13	58.34	35.95	43.98	56.82	22.58	26.84
8	58	2	6	breast cancer	15.9	1	18	3	0	6	226.45	302.87	239.76	71.27	109.48	32.93
9	60	1	0		10.4	0	21	2	0	6	13.74	18.71	11.15	23.09	133.74	7.63
10	74	1	1	HT	43.8	1	23	3.7	0	6	29.21	42.61	11.37	18.64	15.5	28.43
11	54	1	0		2.1	0	24	3.3	0	7	71.75	28.62	71.69	78.47	22.21	96.49
12	53	1	0		0	0	19	3	0	8	28.97	21.96	23.36	36.01	15.79	11.28
13	49	1	0		12.5	0	20	5.7	0	30	34.18	40.78	24.26	38.29	23.35	12.53
14	53	2	0		25	1	25	3	0	3	11.16	23.22	17.31	45.77	48.18	17.8
15	74	2	1	HT	0.1	0	24	4	0	2	49.28	27.83	36.97	13.93	48.83	19.83
16	53	2	0		43.8	1	18	3	2	6	10.8	23.34	25.31	27.48	24.63	16.55
17	42	2	0		8.3	0	19	8	0	35	25.25	83.72	30.15	25.31	56.29	29.14
18	58	2	0		31.8	1	28	5	0	3	27.81	116.56	38.33	32.03	22.57	32.68
19	48	1	0		25	1	23	3	0	6	8.2	13.11	13.19	6.34	38.36	18.88
20	51	2	0		25	1	12	3	0	1	18.17	12.7	5.92	9.84	13.91	51.08
21	53	2	0		18.8	1	26	4.3	1	0	13.51	20.83	20.91	11.16	17.15	10.82
22	46	1	0		0	0	23	2	2	2	61.66	57.93	229.17	65.08	30.11	114.8
23	40	2	0		22.9	1	19	2.3	0	8	16.39	16.66	21.68	19.41	6.73	11.87
24	81	1	1	HT	4.2	0	25	5	0	3	8.99	3.34	10.54	11.79	12.93	25.82
25	46	1	1	HT	31.3	1	15	3	0	2	16.1	7.21	24.54	20.16	21.64	46.55
26	68	2	0		4.5	0	23	5	0	4	11.74	10.8	7.32	12.4	17.68	34.23
27	49	2	0		14.6	1	24	2	1	3	46.06	23.61	27.91	21.98	5.9	56.29
29	51	2	0		31.3	1	23	2.3	0	5	19.84	12.8	10.27	5.41	12.92	10.38
30	38	2	0		8.3	0	22	6.3	0	11	31.89	6.31	17.09	9.31	16.73	29.74
31	54	2	0		6.8	0	21	6.7	0	5	21.51	43.16	46.37	27.65	71.68	44.59
32	55	2	0		14.5	1	17	2	0	4	13.71	10.69	11.02	29.51	8.81	17.68
33	71	2	0		41.7	1	19	3.7	0	6	123.41	72.26	22.03	13.77	44.75	102.87
34	45	2	0		12.5	1	21	3	0	4	16.14	23.01	29.51	16.61	19.53	32.18
35	51	2	0		2.1	0	17	5.7	0	12	22.7	46.88	24.13	51.34	32.59	23.11

LF5	LF6	LF7	LF8	LF before instillation	LF after instillation	LFsd before instillation	LFsd after instillation	HF0	HF1	HF2	HF3
24.62	22.17	26.32	172.94	82.71	54.83	62.51	66.06	14.4	65.25	19.48	13.02
54.65	12.29	16.16	193.33	18.69	69.28	17.87	73.61	34.5	5.3	7.86	9.12
25.49	50.12	24.13	25.76	25.55	27.74	6.69	13.55	9.6	8.37	9.27	2.19
30.48	45.61	30.38	28.99	44.15	32.65	17.43	7.33	40.45	37.44	48.9	44.3
12.85	23.57	35.63	33.54	31.04	28.05	22.13	9.78	23.67	4.89	10.7	30.04
55.4	74.84	58.57	42.13	81.03	61.88	29.57	14.88	34.7	48.77	18.3	31.32
33.69	22.14	36.21	23.09	43.53	28.39	14.94	6.3	26.68	25.62	20.1	28.08
90.8	181.73	154.23	135.05	189.97	118.95	96.34	58.39	87.1	142.79	165.94	43.89
10.22	21.73	12.6	20.34	40.09	14.51	52.56	6.24	10.62	6.88	7.3	8.09
10.82	16.61	24.03	44.03	23.47	24.78	12.58	12.71	24.33	19.22	15.24	19.49
42	17.52	56.22		54.55	53.06	26.83	33.07	34.67	30.67	24.42	5.66
12	31.71	24.69	634.67	25.22	142.87	7.64	275.06	8.34	9.36	11.33	5.2
28.22	25.49	41.01	17.94	32.17	25.04	8	10.87	5.99	8.1	5.74	5.82
18.29	10.31	7.35	34.99	29.13	17.75	16.86	10.74	53.01	20	32.99	11.88
22.29	21.25	23.9	32.28	35.37	23.91	14.95	4.91	14.41	15.57	16.44	21.02
29.4	27.36	27.41	32.98	22.31	26.74	6.61	6.14	8.87	5.6	9.23	8.63
41.59	38.21	18.7	15.26	44.15	28.58	25.6	11.59	21.77	36.15	13.49	23.56
40.82	31.85	25.87	29.46	47.46	32.14	39.06	5.53	8.73	70.97	25.59	13.06
12.74	33.92	34.09		15.84	24.91	12.94	10.8	6.25	9.28	8.79	7.85
17.78	12.19	13.95	10.79	12.11	21.16	4.58	16.93	10.74	8.65	8.49	13.27
13.56	15.81	34.41	16.06	16.71	18.13	4.35	9.34	14.52	10.61	8.23	3.3
18.51	27.02	40.09	31.02	88.79	46.29	79.69	39.08	67.7	78.07	75.87	17.84
12.09	16.53	26.47	21.53	16.17	17.7	5.71	6.29	40.74	43.67	28.08	33.99
9.64	21.13	8.27	17.85	9.52	16.54	3.75	7.5	9.81	5.76	6.07	5.83
35.4	63.93	19.1	71.45	17.93	47.29	6.72	21.2	18.69	17.55	6.34	10.49
7.97	11.44	12.82	8.2	11.99	14.93	3.74	10.99	6.17	2.73	3.81	2.51
21.75	29.75	20.91	41.07	25.09	33.95	14.39	14.89	11.95	14.64	2.5	6.36
34.21	10.1	21.92	16.32	12.25	18.58	5.22	10	24.31	16.86	18.19	14.71
39.96	10.21	15.04	12.71	16.27	21.53	9.91	12.8	50.61	34.76	34.45	33.28
19.76	29.97	53.61	26.33	42.07	34.85	19.54	13.88	55.59	49.19	42.72	47.98
34.5	8.07	5.15	8.47	14.75	14.77	8.44	11.99	12.71	7.45	10.37	18.93
31.16	24.09	21.54	4.37	55.24	36.81	44.36	38.22	114.86	52.63	9.64	9.64
20.07	12.12	17.9	14.64	20.96	19.38	5.51	7.77	7.52	2.75	1.8	0.88
13.6	15.97	12.43	28.38	35.53	18.7	13.06	6.82	15.1	6.47	10.62	14.99

HF4	HF5	HF6	HF7	HF8	HF9	HF before instillation	HF after instillation	HFsd before instillation	HFsd after instillation	LFHF0	LFHF1
11.47	16.21	5.18	11.95	11.51	60.45	24.72	21.06	22.85	22.37	2.32	1.46
9.85	54.29	21	7.1	12.29	186.69	13.33	56.27	11.96	75.18	1.46	1.72
3.05	11.51	3.53	4.67	7.73	5.7	6.5	6.63	3.58	3.14	2.28	2.45
36.5	24.08	39.5	42.44	27	24.79	41.52	31.56	5.13	8.72	1.12	0.65
5.3	9.09	11.35	5.61	18.37	27.08	14.92	14.3	11.36	8.53	1.25	1.71
5.07	5.29	8.27	16.51	16.02	10.05	27.63	11.23	16.63	4.91	1.22	1.35
24.36	25.95	23.85	33.91	24.97	33.3	24.97	28.4	3.05	4.82	2.19	1.4
40.78	51.11	72.91	217.12	197.63	144.65	96.1	136.69	56.84	73.55	2.6	2.12
11.5	7.57	5.57	3.83	7.04	3.91	8.88	5.59	2.06	1.73	1.29	2.72
10.39	14.22	7.79	13.54	18.07	2.69	17.73	11.26	5.22	6.04	1.2	2.22
15.34	48.11	34.03	33.75	31.6		22.15	36.88	11.76	7.57	2.07	0.93
6.2	4.59	10.64	5.57	8.79	69.27	8.09	19.77	2.45	27.78	3.47	2.35
2.85	2.11	7.11	8.63	22.31	10.79	5.7	10.19	1.87	7.49	5.7	5.04
14.38	9.57	36.85	11.41	12.49	33.34	26.45	20.73	16.94	13.21	0.21	1.16
5.28	8.41	16.74	6.48	8.78	13.79	14.54	10.84	5.75	4.26	3.42	1.79
2.76	10.72	11.53	3.14	3.77	6.03	7.02	7.04	2.79	3.89	1.22	4.17
31.68	18.36	14.75	12.9	32.88	25.8	25.33	20.94	8.85	8.3	1.16	2.32
28.95	6.86	6.24	6.87	11.51	7.43	29.46	7.78	24.68	2.13	3.19	1.64
3.19	7.82	9.02	3.14	7.58		7.07	6.89	2.46	2.58	1.31	1.41
13.65	17.72	16.04	4.39	1.84	4.9	10.96	8.98	2.45	7.33	1.69	1.47
8.75	4.79	5.37	7.4	8.05	9.16	9.08	6.96	4.07	1.83	0.93	1.96
23.35	23.9	13.74	13.58	11.41	15.23	52.56	15.57	29.5	4.85	0.91	0.74
28.48	24.48	35.45	24.4	25.09	27.46	34.99	27.38	7.06	4.68	0.4	0.38
8.59	4.91	7.82	3.95	4.05	4.43	7.21	5.03	1.87	1.6	0.92	0.58
6.03	19.73	11.41	7.64	7.65	9.2	11.82	11.12	6.03	5.05	0.86	0.41
1.79	4.11	10.57	9.54	9.98	10.1	3.4	8.86	1.71	2.68	1.9	3.96
4.39	23.28	9.18	8.66	13.22	4.97	7.97	11.86	5.14	7.02	3.86	1.61
21.97	8.88	14.72	16.76	9.55	12.27	19.21	12.43	3.89	3.35	0.82	0.76
30.2	27.1	28.54	35.29	34.78	31.72	36.66	31.49	8	3.65	0.63	0.18
39.73	25.25	22.03	27.26	33.29	26.3	47.04	26.83	6.14	4.12	0.39	0.88
10.97	7.47	7.55	10.54	5.39	4.7	12.09	7.13	4.27	2.28	1.08	1.44
47.24	75.62	10.13	12.34	9.45	7.69	46.8	23.05	43.09	29.44	1.07	1.37
2.66	9.82	1.93	3.43	5.99	4.11	3.12	5.06	2.57	3.04	2.15	8.36
14.99	5.97	15.09	18.06	15.28	16.52	12.43	14.18	3.84	4.74	1.5	7.25

LFHF2	LFHF3	LFHF4	LFHF5	LFHF6	LFHF7	LFHF8	LFHF9	LFHF before instillation	LFHF after instillation	LFHFsd before instillation	LFHFsd after instillation
9.29	6.05	2.16	1.73	4.75	1.86	2.29	2.86	4.26	2.7	3.34	1.23
1.3	1.7	0.85	1.29	2.6	1.73	1.31	1.04	1.4	1.59	0.36	0.62
3.48	9	10.95	1.15	7.22	10.74	3.12	4.52	5.63	5.35	4.05	3.74
0.86	1.62	1.02	1.15	0.77	1.07	1.13	1.17	1.05	1.06	0.36	0.16
1.29	2.12	7.51	3.81	1.13	4.2	1.94	1.24	2.78	2.47	2.67	1.45
4.19	3.32	22.94	14.83	6.7	4.53	3.66	4.19	6.61	6.78	9.22	4.64
2.19	2.02	0.93	1.03	1.41	0.65	1.45	0.69	1.75	1.05	0.56	0.38
1.44	1.62	2.68	0.64	1.25	0.84	0.78	0.93	2.09	0.89	0.56	0.23
1.53	2.86	11.63	1.01	1.83	5.67	1.79	5.21	4.01	3.1	0.57	6.66
0.75	0.96	1.49	2	1.39	1.23	1.33	16.37	1.32	4.46	5.42	0.6
2.94	13.86	1.45	2.01	1.23	0.52	1.78	1	4.25	1.31	2.01	3.21
2.06	6.93	2.55	2.46	1.13	5.7	2.81	9.16	3.47	4.25	1.53	1.76
4.23	6.58	8.2	5.94	3.97	2.95	1.84	1.66	5.95	3.27	1.67	0.54
0.52	3.85	3.35	1.86	0.5	0.9	0.59	1.05	1.82	0.98	3.38	0.71
2.25	0.66	9.26	2.36	1.33	3.28	2.72	2.34	3.47	2.41	0.58	1.16
2.74	3.19	8.94	1.54	2.55	8.7	7.27	5.47	4.05	5.11	0.93	1.55
2.24	1.07	1.78	1.59	2.82	2.96	0.57	0.59	1.71	1.71	6.51	9.33
1.5	2.45	0.78	4.76	6.54	4.63	2.25	3.97	1.91	4.43	4.83	4.02
1.5	0.81	12.03	2.41	1.41	10.79	4.5	1	3.41	4.02	4.83	4.02
0.7	0.74	1.02	2.88	1.11	2.77	7.58	2.2	1.12	3.31	0.44	1.28
2.54	3.38	1.96	2.26	2.52	2.14	4.27	1.75	2.15	2.59	1.32	1.4
3.02	3.65	1.29	4.8	1.35	1.99	3.51	2.04	1.92	2.74	0.59	1.87
0.77	0.57	0.24	0.48	0.34	0.68	1.05	0.78	0.47	0.67	1.56	2.99
1.73	2.02	1.51	5.26	1.23	5.35	2.04	4.03	1.35	3.58	3.27	3.28
3.87	1.92	3.59	2.36	3.1	8.37	2.5	7.77	2.13	4.82	4.01	2.68
1.92	4.95	9.89	8.33	0.75	1.2	1.28	0.81	4.52	2.48	0.18	0.75
11.19	3.46	1.34	2.42	2.37	3.43	1.58	8.26	4.29	3.61	0.19	0.49
0.56	0.37	0.59	1.17	2.32	0.6	2.3	1.33	0.62	1.54	0.55	0.39
0.5	0.28	0.55	1.1	1.4	0.29	0.43	0.4	0.43	0.72	0.31	1.53
1.09	0.58	1.8	1.77	0.9	1.1	1.61	1	0.95	1.27	0.52	0.95
1.06	1.56	0.8	2.37	4.57	0.77	0.96	1.8	1.19	2.09	5.06	7.11
2.29	1.43	0.95	1.36	3.08	1.95	2.28	0.57	1.42	1.85	6.88	3.17
16.39	18.89	7.36	3.28	10.4	3.53	2.99	3.56	10.63	4.75	6.88	3.17
2.27	3.42	2.17	3.87	0.9	0.88	0.81	1.72	3.32	1.64	2.3	1.3
